# Cancer Immunotherapy by Targeting IDO1/TDO and Their Downstream Effectors

**DOI:** 10.3389/fimmu.2014.00673

**Published:** 2015-01-12

**Authors:** Michael Platten, Nikolaus von Knebel Doeberitz, Iris Oezen, Wolfgang Wick, Katharina Ochs

**Affiliations:** ^1^Neurology Clinic, University Hospital Heidelberg and National Center for Tumor Diseases, Heidelberg, Germany; ^2^DKTK Clinical Cooperation Unit Neuroimmunology and Brain Tumor Immunology, German Cancer Research Center (DKFZ), Heidelberg, Germany; ^3^DKTK Clinical Cooperation Unit Neurooncology, German Cancer Research Center (DKFZ), Heidelberg, Germany

**Keywords:** IDO, TDO, AhR, tumor immunity, tryptophan metabolism

## Abstract

The tryptophan (TRP) to kynurenine (KYN) metabolic pathway is now firmly established as a key regulator of innate and adaptive immunity. A plethora of preclinical models suggests that this immune tolerance pathway – driven by the key and rate-limiting enzymes indoleamine-2,3-dioxygenase and TRP-2,3-dioxygenase – is active in cancer immunity, autoimmunity, infection, transplant rejection, and allergy. Drugs targeting this pathway, specifically indoleamine-2,3-dioxygenase, are already in clinical trials with the aim at reverting cancer-induced immunosuppression. In the past years, there has been an increase in our understanding of the regulation and downstream mediators of TRP metabolism, such as the aryl hydrocarbon receptor as a receptor for KYN and kynurenic acid. This more detailed understanding will expand our opportunities to interfere with the pathway therapeutically on multiple levels. Here, we discuss the perspective of targeting TRP metabolism at these different levels based on reviewing recent insight into the regulation of TRP metabolism and its downstream effectors.

## Introduction

The catabolism of the essential amino acid tryptophan (TRP) is a central pathway maintaining the immunosuppressive microenvironment in many types of cancers. The classic concept proposes that tumor cells or myeloid cells in the tumor microenvironment or draining lymph nodes express high levels of indoleamine-2,3-dioxygenase 1 (IDO1), which is the first and rate-limiting enzyme in the degradation of TRP. This enzymatic activity results in the depletion of TRP in the local microenvironment and subsequent inhibition of T cell responses. T cells sense low TRP levels via uncharged tRNAs and subsequently activating the kinase general control non-derepressible 2 (GCN2) and initiating an amino acid starvation response resulting in cell cycle arrest and cell death. This rather non-specific metabolic pathway exerts immunosuppression in the local microenvironment as T cells are particularly sensitive to low TRP levels. This IDO1-centered concept is supported by numerous preclinical studies in models of tumor immunity, autoimmunity, infection, and allergy. More recent preclinical studies, however, propose an alternative route of TRP degradation in tumors via the enzyme TRP-2,3-dioxygenase 2 (TDO), which was previously believed to be liver- and neuron-specific. Tumor cells and possibly specialized myeloid cells may express and catabolize TRP via TDO instead of or in addition to IDO1. Thus, TDO may represent an additional target for cancer immunotherapy, while both enzymes ought to employ identical downstream effectors, such as GCN2.

The effector function of GCN2 in the context of cancer immunity, however, is less well understood and established. In addition, several studies have proposed that immunosuppression by TRP degradation is not solely a consequence of lowering local TRP levels but also of accumulating high levels of TRP metabolites. This alternative or additional concept is supported by studies demonstrating that T cell responses are inhibited by TRP metabolites, mainly by binding to the aryl hydrocarbon receptor (AHR), a cytoplasmic transcription factor, previously believed to be solely responsible for detoxification of polyaromatic hydrocarbons. The importance of the AHR in regulating autoimmunity and tumor immunity is supported by preclinical studies and analyses of human tumor tissue demonstrating that binding of the TRP metabolite kynurenine (KYN) to the AHR results in reprograming the differentiation of naïve CD4+ T-helper (Th) cells favoring a regulatory T cells phenotype (Treg) while suppressing the differentiation into interleukin-17 (IL-17)-producing Th (Th17) cells. Notably, activation of the AHR also results in promoting a tolerogenic phenotype on dendritic cells (DC). The AHR seems to be required for the induction of IDO in dendritic cells (1) and stimulation with the poisonous AHR-agonist 2,3,7,8-tetrachlorodibenzo-p-dioxin (TCDD) was shown to induce IDO expression in dendritic cells (DC) (2), suggesting a feed-forward loop of immunosuppressive TRP metabolism. The role of the AHR on CD8+ effector T cells is less well understood.

While first clinical trials with IDO1 inhibitors are underway, this review aims at putting the recent advances in understanding the immunobiology of TRP catabolism via IDO1/TDO in therapeutic perspective for cancer immunotherapy.

## Multiple Therapeutic Targets in Tryptophan Catabolism

Current preclinical studies suggest that the opportunity to interfere with immunosuppressive TRP catabolism goes well beyond restoring TRP levels by inhibiting the enzymatic activity of IDO1. First, tumors may catabolize TRP by alternative enzymatic routes such as TDO. A survey of cancer cell lines indicates that 16% of tumor cell lines are IDO1 positive, while 19% are TDO positive and 15% express both TDO and IDO1 (3). These observations suggest that targeting TDO may complement IDO1 inhibition. Remarkably, IDO1 inhibitors available to date do not cross-inhibit TDO and vice-versa, probably due to low sequence homology of these two enzymes despite similar enzymatic properties. Second, several studies have indicated that IDO1 is intricately linked to an oncogenic signaling pathway, opening new therapeutic avenues to inhibit IDO on a more upstream transcriptional or translational level. Promising upstream targets of IDO1 include KIT, signal transducer and activator of transcription 3 (STAT3), and the tumor suppressor Bin1 (4–6). Also, there is new evidence of IDO1 promoting self-tolerance via non-enzymatic signaling pathways involving transforming growth factor-β (TGF-β) (7). This non-enzymatic activity of IDO1 would only be targeted by strategies interfering with upstream pathways regulating IDO1 transcription and/or translation. Third, low TRP levels, which are believed to mediate to a relevant extent the immunosuppressive activity of IDO1, are sensed by the stress kinase GCN2 activated in T cells in low TRP conditions. The specific pathways transducing the immunosuppressive signals by activated GCN2 in T cells are yet to be identified. Alternatively or in addition, low TRP levels may be sensed by the signaling complex mammalian target of rapamycin (mTOR), which may provide a TRP sufficiency signal not only in cancer cells but also in T cells. Fourth, TRP metabolites such as KYN, 3-hydroxy-kynurenine (3-HK), and kynurenic acid (KA), which may accumulate in the local microenvironment due to high activity of IDO1 and/or TDO, actively suppress T cell responses. These activities are – at least in part – mediated by binding to the AHR, which is – among other effects – involved in the control of differentiation and activation of Tregs. The role of the orphan G protein-coupled receptor GPR35 as a receptor for KYN and KA in immunity is currently unclear. Fifth, transcellular transport systems responsible for shuttling TRP and its metabolites in and out of its target cell include several promiscuous but possibly other more specific transporters whose function in regulating immune responses are not well understood, but, which may serve as potential therapeutic targets, for instance, with the aim at maintaining high intracellular TRP levels in immune effector cells despite low extracellular TRP levels.

**Figure 1:**
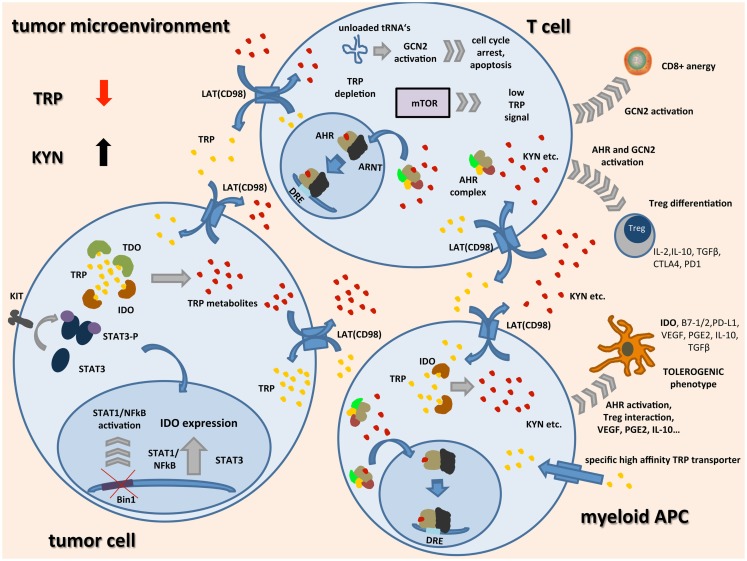
**Schematic representation of TRP catabolism in the tumor microenvironment:** expression of indoleamine-2,3-dioxygenase (IDO) and tryptophan-2,3-dioxygenase (TDO) by tumor cells and infiltrating immune cells leads to depletion of the essential amino acid tryptophan (TRP) while metabolites such as kynurenine (KYN) accumulate. Upon binding of TRP catabolites, the aryl hydrocarbon receptor (AHR) translocates to the nucleus, dimerizes with AHR nuclear translocator (ARNT), and induces the expression of its target genes by binding to dioxin-responsive elements (DRE). In tumor cells, expression of IDO seems to be linked to oncogenic signaling pathways such as loss of tumor suppressor gene Bin1 with subsequent STAT1 and NFκB activation, and STAT3 activation through mutated receptor tyrosine kinases such as KIT. IDO expression in APCs seems to be associated with a regulatory phenotype and is among others driven by AHR activation, cell–cell contact with tumor cells, and Tregs through PD-1/PD-L1 or B7/CTLA4 interaction (not shown), and soluble factors such as PGE2, IFNγ, VEGF, and IL-10. In most cell types, transcellular TRP transport is handled by light chain glycoprotein-associated amino acid transporters (LAT) functioning as TRP/KYN antiporters. IDO expressing tumor cells and APCs additionally express high-affinity TRP transporters leading to a shift of remaining TRP away from highly TRP-dependent T cells. There, general control non-derepressible 2 (GCN2) activation through accumulation of unloaded tRNAs, mTOR signaling, and AHR activation lead to inhibition of CD8+ effector T cells while differentiation of Tregs is enhanced. These Tregs together with tolerogenic APCs further inhibit an effective anti-tumor immune response by release of immunosuppressive cytokines and inhibitory cell surface proteins.

Conceptually, these five distinct hubs may serve as potential therapeutic targets interfering with immunosuppressive TRP catabolism in the context of cancer and possibly other immune-mediated diseases associated with an activation of TRP metabolism (Figure 1). The potential therapeutic opportunities and challenges associated with these hubs are discussed in the following chapters.

## Targeting IDO1: Finding the Right Combination Partner

IDO1 is now firmly established target of drug discovery in cancer immunotherapy. The first IDO1-inhibitor, 1-methyl-TRP, is a mixture of the two racemic isoforms 1-methyl-l-TRP (1-l-MT) and 1-methyl-d-TRP (1-d-MT). While 1-l-MT is the classic non-competitive inhibitor of IDO1, 1-d-MT has been suggested to be less active in inhibiting IDO1 (8), but showing higher potency in reversing IDO-mediated T cell suppression (9, 10). 1-d-MT is being developed clinically as an IDO-inhibitor (indoximod, NLG8189) for the treatment of several cancers with the aim at reversing cancer-associated immune suppression. Reversal of tumor-associated immune suppression by 1-d-MT appears to be dependent on host IDO1 expression in preclinical models (9). In addition or alternatively to direct IDO inhibition, 1-d-MT may interfere with transcellular TRP transport (11), thereby providing via mTOR a TRP sufficiency signal to the cell (12) and by additional off-target effects (13). As 1-l-MT is principally capable of exerting the same effects, it is not yet entirely clear why 1-d-MT is more effective in restoring T cell activity under physiological conditions (12). 1-d-MT was reported to preferentially target IDO2 (8). IDO2 is an IDO-related enzyme with a different expression pattern. The physiological relevance of IDO2 particularly in humans remains unclear (8, 10, 14); further studies are required to determine whether it may serve as a suitable target for cancer immunotherapy.

In addition to directly inhibiting IDO enzymatic activity, second-generation IDO1 inhibitors such as INCB024360 may have a more favorable pharmacokinetic profile. While phase I clinical trials with these orally available compounds have demonstrated safety (15) and indicated biological efficacy based on serum parameters demonstrating reversal of TRP depletion and KYN accumulation, it is questionable whether these compounds will be effective by themselves. The major challenge for designing future clinical trials will be to find the appropriate combination partner. In this respect, preclinical studies have provided valuable insight into potential strategies to amplify the efficacy of IDO1 inhibitors. Initial experiments have applied 1-MT in combination with chemotherapy (6). Consequently, clinical phase I trials have combined indoximod with chemotherapy (16–18). Based on early observations that IDO1 is induced in DCs following ligation of B7 molecules by cytotoxic T-lymphocyte-associated protein 4 (CTLA4) (19–21), a recent preclinical study suggested that IDO1 is a critical resistance mechanism attenuating the efficacy of anti-CTLA4 antibodies in cancer immunotherapy. Interestingly, in this study this crucial role as a resistance factor is not restricted to anti-CTLA4 antibodies but also antibodies to programed cell death 1 (PD-1) and programed death-ligand 1 (PD-L1) (22). Trials combining indoximod (23) or INCB024360 (24) with the anti-CTLA4 antibody ipilimumab in patients with melanoma are underway. Conceptually and also supported by preclinical studies, IDO1 inhibition may enhance the efficacy of active cancer vaccines as it may break cancer-induced tolerance. Two phase II studies are currently evaluating this combination approach (25, 26).

It has been shown that prostaglandin E2 (PGE2) expression in a cancer setting induces regulatory T cells, promotes T cell anergy through direct effects on T cells and indirect effects via antigen presenting cells (APCs) (27), thus, like IDO, shifting the immune system toward a tolerogenic phenotype and promoting tumor progression. Cyclooxygenase 2 (COX2), key enzyme in the production cascade of PGE2, like IDO, is expressed at low levels by most somatic cells but is upregulated in many types of cancer cells and tumor-infiltrating APC (27). Interestingly, PGE2 may be part of the immunosuppressive KYN-AHR feed-forward loop by driving IDO and TDO [Ref. (28) and unpublished observations]. It seems reasonable that suppression of anti-tumor immunity via PGE2 and IDO are not separately working mechanisms but rather contribute synergistically to tumor immune evasion. Thus, a combinatorial approach with IDO- and COX2-inhibitors might be an interesting option to break suppression of anti-cancer immunity.

Besides finding the right combination partner, a future challenge will certainly be the identification of cancer types and patients who will benefit from an IDO1 inhibitory approach. Current trials do not select patients based on IDO1 expression in tumor tissue or assessment of systemic IDO1 activity by analysis of TRP and its metabolites in patients’ serum. It is conceivable that this approach may be most successful in cancer types, which are immunogenic *per se*, such as malignant melanoma. Another challenge will certainly be the assessment of possible escape strategies that cancers may develop. There is essentially no data on such potential evasion strategies in preclinical studies.

## Non-Enzymatic Targeting of IDO1

In the past years, it has become increasingly clear that IDO1 is part of an oncogenic signature in cancer supporting the concept that cell-autonomous pathways driving cancer cells are intricately linked to an immunosuppressive phenotype (29). For instance, IDO1 is controlled by the tumor suppressor gene *Bin1* encoding the Myc-box-encoding protein 1. Loss of Bin1 in tumors results in transcriptional upregulation of IDO1 via STAT1 and *nuclear factor* (NF)-kB and subsequent escape from T cell-dependent anti-tumor immunity (6). In addition, a recent study suggests that IDO1 is also driven by oncogenic KIT signaling in gastrointestinal stromal tumors (GIST). Treatment of experimental tumors with imatinib resulted in the reversal of IDO1-mediated immunosuppression and thus activation of T effector cells and suppression of Tregs, which was dependent on IDO1, which was suppressed by imatinib (4). These data suggest that this targeted agent may derive its remarkable clinical effects in this tumor entity from its profound immunological effects and advocate for conducting preclinical studies in tumor models involving immunocompetent hosts. Based on the link between CTLA4 and IDO1, a rational therapeutic consequence of this observation is to combine imatinib with an anti-CTLA4 approach in GIST, which is currently tested in a clinical trial (30). IDO1 expression, which is classically induced by proinflammatory cytokines, is tightly controlled by STAT molecules. These pathways are also activated by oncogenic signaling pathways. For instance, STAT3, which is activated by KIT and also growth factors such as epidermal growth factor (EGF) and cytokines such as IL-6 transcriptionally activates IDO1 by binding to its promoter (5). As new compounds targeting, for instance, activated STAT3 are now in clinical trials (31), there is a great opportunity for investigating, whether the beneficial and potentially immunostimulatory effects of these agents are also dependent on IDO1. The same mechanisms that mediate transcriptional or translational activation of IDO1 may affect its stability. For instance, the STAT antagonist suppressor of cytokine signaling (SOCS) 3 promotes the active protein to bind IDO1 and promote its proteasomal degradation (32). This intriguing observation further strengthens the rational to interfere with IDO protein expression in addition or alternatively to IDO1 enzyme inhibition. Along this line are preclinical and clinical studies targeting IDO1 by a peptide vaccine (33). Here, it will be fascinating to see how elimination of IDO-expressing cells will alter the immunosuppressive tumor microenvironment and allow for a more efficient anti-tumor immunity. We are only beginning to understand the complex network interacting with IDO1. This complexity is even increased by recent observations that IDO1 may act as a signaling molecule mediating or sustaining immune tolerance independent of its enzymatic activity (7). This observation further supports the concept that IDO1 in cancer immune therapy ought to be targeted not only at the enzymatic level.

## Targeting TDO: Aiming at a Liver Enzyme

With two recent studies demonstrating that TRP metabolism via TDO represents an alternative route to IDO1 activity employed by tumors (3, 34), there is an interest in pharmacological targeting of TDO for cancer immunotherapy. This interest is fueled by the fact that currently available IDO1 inhibitors do not target TDO. Based on the lead structure of 68OC91 (35), the indole LM10 has recently been developed with a more favorable pharmacokinetic profile (36). One concern of systemic TDO inhibition is safety. In contrast to IDO1, TDO is strongly expressed constitutively in the liver, where it is believed to be responsible for maintaining systemic TRP levels, and – albeit at lower levels – in neurons. There are two lines of evidence that TDO may be targeted safely with a specific inhibitor: currently, preclinical studies have not documented relevant liver toxicity using LM10 (3) and TDO-deficient mice develop normally and display an unremarkable phenotype except for an increased neurogenesis and a less anxious phenotype (37). The latter may be due to increased levels of 5-hydroxy-TRP (5-HTP) in the hippocampus. While these may be beneficial effects in some diseases, CNS-specific side effects ought to be closely monitored in future preclinical and clinical studies. It also needs to be taken into consideration that systemic TDO inhibition will result in increased levels of TRP metabolites such as KYN due to increased availability of TRP for IDO1 as suggested by the TDO-deficient mice. If TRP metabolites are relevant in mediating the immunosuppressive and tolerogenic effects of TRP catabolism in cancer, a dual approach needs to be considered to combine an anti-TDO strategy with inhibitors of KYN. In addition to identifying novel TDO inhibiting compounds, it is logical to test – based on the experiences with IDO1 – existing anti-cancer compounds for their potential to inhibit TDO (38). Here, the understanding of the signaling pathways driving constitutive TDO expression in tumor cells is key to interfere with this pathway. First studies aiming at deciphering these pathways have just been published and reveal fundamental differences in the regulation of TDO in cancer cells versus untransformed cells (39).

## Targeting AHR: Challenging a Promiscuous Receptor

Several metabolites of TRP including photoproducts such as 6-formylindolo-[3,2-b]carbazole (FICZ) (40), bacterial products such as indole-3-aldehyde, phenazines, and naphthoquinones (41, 42), and plant products such as indoles, flavonoids, and polyphenoles (43) have been shown to be ligands of the AHR. Since the discovery that AHR-deficient mice are prone to autoimmunity (44, 45) and that mice expressing a constitutively active AHR are prone to develop tumors (46), it has been speculated that endogenous TRP metabolites are responsible for inducing AHR-mediated tolerance. Indeed, a recent study has demonstrated that TRP metabolites produced by IDO1 and/or TDO induce tolerance to bacterial products via the AHR (47). This study is important as it supports the concept that endogenous TRP levels produced by IDO1/TDO accumulate at levels sufficient to activate the AHR. Of note, the IC50 of KYN and KA for the AHR is in the low micromolar range (34, 48). As tumors produce high levels of these metabolites in the order of 30–50 μM, it comes as no surprise that the levels are sufficient to activate the AHR (34). As preclinical studies also suggest that the AHR is responsible for mediating – at least in part – the immunosuppressive effects of cancer-derived TRP metabolites (34, 48), the AHR represents a logical pharmaceutical target for cancer immunotherapy. Several challenges have to be met in developing AHR antagonists to cancer immunotherapy: first, the AHR is a promiscuous receptor binding several structurally diverse molecules with different affinity. Many studies evaluating AHR ligands rely on luciferase assays measuring the transcriptional activity of dioxin responsive elements (DRE) bound by the AHR. Often times, however, compounds, which induce AHR-dependent DRE activity, are not direct AHR ligands but rather facilitate its activity as a transcription factor. Here, the fact that it has not been possible to date to crystallize the AHR for receptor–ligand interaction studies has been an important hurdle in the development of drugs binding to the AHR. Clearly, due to its promiscuity, screens based on cellular luciferase assays have to cope with a high hit rate. On the other hand, there are compounds already available with AHR antagonistic activity. Whether these compounds are capable of blocking all AHR ligands including TRP metabolites is unclear. Second, there are safety concerns with respect to pharmacological AHR inhibition. While AHR knock-out mice develop normally and display only mild immunological aberrations including deficiency of specific resident immune cells in the gut and skin, challenge of these mice with drugs, which are metabolized via the AHR, may result in toxicity, which would also be observed in patients treated with AHR antagonists. Third, it is not entirely clear, which cellular and molecular mechanisms are involved in AHR-mediated tolerance to tumors. As AHR activation has been shown to induce a tolerogenic phenotype in DCs and modulate Treg differentiation, it may well be possible that the AHR acts at multiple levels of the immune compartment. Clearly, further studies are warranted to clarify these challenges before moving AHR antagonistic strategies to the clinic.

## Targeting GCN2: Interfering with Multiple Pathways

The classical effector pathway of immunosuppressive TRP metabolism involves the activation of the stress kinase GCN2. GCN2 is activated by uncharged tryptophanyl tRNAs accumulating in conditions of low TRP levels. Interestingly, although GCN2 has been suggested as a key mediator of the T cell suppressive effects of low TRP conditions (49), remarkably little is known about how GCN2 regulates T cell function. While in low TRP environments, GCN2 in CD8+ effector T cells is important for induction of anergy following TCR stimulation (49), in CD4+ T cells GCN2 appears to be important for the expansion and activation of regulatory T cells (50). Of note, GCN2 is also expressed in DCs where it appears to aid antigen presentation by regulating autophagy. While it is in principle conceivable that GCN2 may be a therapeutic target in the immunosuppressive TRP pathway, there are several challenges ahead: first, GCN2 is ubiquitously expressed and important in regulating response not only to amino acid deprivation but also to other forms of cellular stress including UV irradiation. Again, GCN2-deficient mice are remarkably normal but display abnormalities in regulating body weight owing to a crucial role of neuronal GCN2 in regulating eating behavior in response to nutritional cues. As cellular stress is a crucial hallmark of cancer affecting tumor cells and the tumor stroma, further studies are required to delineate the role of GCN2 in this context. Also remarkably, there is no evidence that host GCN2 is relevant in regulating tumor immunity in transplantable or spontaneous syngeneic tumor models not requiring adoptive transfer of antigen-specific T cells. Clearly, these studies have to be performed, also to enable the identification of key signaling pathways involved in GCN2-mediated alteration of T cell function in response to tumors with active TRP metabolism.

## TRP Transport Pathways – An Underrecognized Variable

Induction of TRP dioxygenase enzyme activity in general may result in a dramatic drop in extracellular TRP levels. The observation, for instance, that IDO-mediated immune suppression can be reversed in certain paradigms by supplementing TRP has led to the hypothesis that the depletion of extracellular TRP suppresses T cell function (51). This hypothesis, however, is based on the presumption that extra- and intracellular TRP pools are equilibrated. Transmembrane TRP transport is chiefly regulated by two distinct systems: the T-system (T-type amino acid transporter, TAT) and the l-system (light chain glycoprotein-associated amino acid transporter, LAT). Most cell types use the l-system to transport TRP across the cell membrane. The placenta, for instance, which is an organ with high IDO activity, solely relies on the l-system to achieve TRP influx (52). The l-system is a heterodimeric transmembrane receptor consisting of a heavy chain (4F2hc, CD98hc) and a light chain (LAT1 or LAT2), the latter representing the catalytic subunits. Interestingly, the l-system is identical with CD98, a cell surface receptor originally identified as an antigen expressed on the cell surface of tumor cells and activated T cells (53). CD98hc interacts with and modulates the cell adhesion properties of integrins (54). System L is commonly overexpressed in tumor cells and seems to be the main route for transcellular TRP transport in T cells (55). Myeloid APCs have been shown to express an additional high-affinity TRP transport mechanism (55) thus being able to take up TRP efficiently in a low TRP containing microenvironment. We have previously shown that System L functions as a TRP/KYN antiport system using a FRET-based TRP sensor (56). While T cells respond to low extracellular TRP levels with growth arrest and anergy, IDO-expressing tumor cells and myeloid APCs might maintain sufficient intracellular TRP levels through KYN/TRP exchange and high-affinity TRP transport. Hence, under TRP depleting conditions, such as cancer, it seems likely that T cells are more affected by TRP starvation and TRP is efficiently being shifted toward TRP consuming cells. The l-system not only binds TRP but also structurally related molecules. The commonly used IDO-inhibitor 1-methyltrypophan (1-MT), for instance, binds LAT1 in breast cancer cells and suppresses TRP influx and enhances TRP efflux (11).

By employing the same transcellular transport mechanisms, 1-d-MT may act as a TRP mimetic, as outlined above, and provide an intracellular TRP sufficiency signal maintaining mTOR activity also in T cells, thus restoring their activity (12). Whether cancer cells rely on TRP metabolism to maintain their NAD levels or whether this pathway represents a rescue system when *de novo* NAD synthesis is not sufficient to provide energy under certain circumstances has not been analyzed.

Collectively, these observations indicate that transmembrane TRP transport by the l-system may play a fundamental role in regulating T cell responses in low TRP environments.

Transcellular transport systems of TRP and its metabolites may thus offer an additional, yet underrecognized potential to influence the consequences of immunosuppressive TRP metabolism at the T cell level to revert cancer-associated immune suppression.

## Summary

Recent advances in understanding the regulation as well as the cellular and molecular targets of TRP metabolism have expanded the opportunity to interfere with this pathway well beyond inhibiting IDO. TDO is actively pursued as a target and multiple approved drugs have been shown to interfere with IDO expression in cancer. With our increased knowledge, future therapeutic strategies will have to take downstream targets such as the AHR but also TRP transport mechanisms into consideration, particularly as these may be more easily and specifically targeted. At the same time, preclinical studies have made clear that such pathway inhibitors may not be active enough as a stand-alone therapeutic approach. Thus, the rational combination with already available and/or yet to be identified immunomodulatory strategies such as cancer vaccines or checkpoint inhibitors based on thorough basic research is warranted. Continuing basic research on this highly conserved and versatile pathway will expand not only our view on its pathophysiological relevance but will also open novel therapeutic avenues for defining novel therapeutic targets for diseases associated with an aberrant immune tolerance, such as cancer, autoimmunity, allergy, transplantation rejection, and infection.

## Conflict of Interest Statement

The authors declare that the research was conducted in the absence of any commercial or financial relationships that could be construed as a potential conflict of interest.

## References

[1] Nguyen NT Kimura A Nakahama T Chinen I Masuda K Nohara K Aryl hydrocarbon receptor negatively regulates dendritic cell immunogenicity via a kynurenine-dependent mechanism. Proc Natl Acad Sci U S A (2010) 107:19961–6. 10.1073/pnas.1014465107 21041655 PMC2993339

[2] Vogel CF Goth SR Dong B Pessah IN Matsumura F. Aryl hydrocarbon receptor signaling mediates expression of indoleamine 2,3-dioxygenase. Biochem Biophys Res Commun (2008) 375:331–5. 10.1016/j.bbrc.2008.07.156 18694728 PMC2583959

[3] Pilotte L Larrieu P Stroobant V Colau D Dolusic E Frederick R Reversal of tumoral immune resistance by inhibition of tryptophan 2,3-dioxygenase. Proc Natl Acad Sci U S A (2012) 109:2497–502. 10.1073/pnas.1113873109 22308364 PMC3289319

[4] Balachandran VP Cavnar MJ Zeng S Bamboat ZM Ocuin LM Obaid H Imatinib potentiates antitumor T cell responses in gastrointestinal stromal tumor through the inhibition of Ido. Nat Med (2011) 17:1094–100. 10.1038/nm.2438 21873989 PMC3278279

[5] Litzenburger UM Opitz CA Sahm F Rauschenbach KJ Trump S Winter M Constitutive IDO expression in human cancer is sustained by an autocrine signaling loop involving IL-6, STAT3 and the AHR. Oncotarget (2014) 5:1038–51. 24657910 10.18632/oncotarget.1637 PMC4011581

[6] Muller AJ DuHadaway JB Donover PS Sutanto-Ward E Prendergast GC. Inhibition of indoleamine 2,3-dioxygenase, an immunoregulatory target of the cancer suppression gene Bin1, potentiates cancer chemotherapy. Nat Med (2005) 11:312–9. 10.1038/nm1196 15711557

[7] Pallotta MT Orabona C Volpi C Vacca C Belladonna ML Bianchi R Indoleamine 2,3-dioxygenase is a signaling protein in long-term tolerance by dendritic cells. Nat Immunol (2011) 12:870–8. 10.1038/ni.2077 21804557

[8] Lob S Konigsrainer A Schafer R Rammensee HG Opelz G Terness P. Levo- but not dextro-1-methyl tryptophan abrogates the IDO activity of human dendritic cells. Blood (2008) 111:2152–4. 10.1182/blood-2007-10-116111 18045970

[9] Hou DY Muller AJ Sharma MD DuHadaway J Banerjee T Johnson M Inhibition of indoleamine 2,3-dioxygenase in dendritic cells by stereoisomers of 1-methyl-tryptophan correlates with antitumor responses. Cancer Res (2007) 67:792–801. 10.1158/0008-5472.CAN-06-2925 17234791

[10] Metz R Duhadaway JB Kamasani U Laury-Kleintop L Muller AJ Prendergast GC. Novel tryptophan catabolic enzyme IDO2 is the preferred biochemical target of the antitumor indoleamine 2,3-dioxygenase inhibitory compound d-1-methyl-tryptophan. Cancer Res (2007) 67:7082–7. 10.1158/0008-5472.CAN-07-1872 17671174

[11] Travers MT Gow IF Barber MC Thomson J Shennan DB. Indoleamine 2,3-dioxygenase activity and l-tryptophan transport in human breast cancer cells. Biochim Biophys Acta (2004) 1661:106–12. 10.1016/j.bbamem.2003.12.004 14967480

[12] Metz R Rust S Duhadaway JB Mautino MR Munn DH Vahanian NN IDO inhibits a tryptophan sufficiency signal that stimulates mTOR: a novel IDO effector pathway targeted by d-1-methyl-tryptophan. Oncoimmunology (2012) 1:1460–8. 10.4161/onci.21716 23264892 PMC3525601

[13] Opitz CA Litzenburger UM Opitz U Sahm F Ochs K Lutz C The indoleamine-2,3-dioxygenase (IDO) inhibitor 1-methyl-d-tryptophan upregulates IDO1 in human cancer cells. PLoS One (2011) 6:e19823. 10.1371/journal.pone.0019823 21625531 PMC3098827

[14] Meininger D Zalameda L Liu Y Stepan LP Borges L McCarter JD Purification and kinetic characterization of human indoleamine 2,3-dioxygenases 1 and 2 (IDO1 and IDO2) and discovery of selective IDO1 inhibitors. Biochim Biophys Acta (2011) 1814:1947–54. 10.1016/j.bbapap.2011.07.023 21835273

[15] Soliman HH Antonia S Sullivan D Vanahanian N Link C Overcoming tumor antigen anergy in human malignancies using the novel indeolamine 2,3-dioxygenase (IDO) enzyme inhibitor, 1-methyl-d-tryptophan (1MT). ASCO Meet Abstr (2009) 27:3004.

[16] Jackson E Dees EC Kauh JS Harvey RD Neuger A Lush R A phase I study of indoximod in combination with docetaxel in metastatic solid tumors. ASCO Meet Abstr (2013) 31:3026.25327557

[17] Jackson E Minton SE Ismail-Khan R Han H Neuger A Antonia S A phase I study of 1-methyl-d-tryptophan in combination with docetaxel in metastatic solid tumors. ASCO Meet Abstr (2012) 30:TS2620.

[18] Zakharia Y Johnson TS Colman H Vahanian NN Link CJ Kennedy E A phase I/II study of the combination of indoximod and temozolomide for adult patients with temozolomide-refractory primary malignant brain tumors. ASCO Meet Abstr (2014) 32:TS2107.

[19] Fallarino F Grohmann U Hwang KW Orabona C Vacca C Bianchi R Modulation of tryptophan catabolism by regulatory T cells. Nat Immunol (2003) 4:1206–12. 10.1038/ni1003 14578884

[20] Grohmann U Orabona C Fallarino F Vacca C Calcinaro F Falorni A CTLA-4-Ig regulates tryptophan catabolism in vivo. Nat Immunol (2002) 3:1097–101. 10.1038/ni846 12368911

[21] Munn DH Sharma MD Mellor AL. Ligation of B7-1/B7-2 by human CD4(+) T cells triggers indoleamine 2,3-dioxygenase activity in dendritic cells. J Immunol (2004) 172:4100–10. 10.4049/jimmunol.172.7.4100 15034022

[22] Holmgaard RB Zamarin D Munn DH Wolchok JD Allison JP. Indoleamine 2,3-dioxygenase is a critical resistance mechanism in antitumor T cell immunotherapy targeting CTLA-4. J Exp Med (2013) 210:1389–402. 10.1084/jem.20130066 23752227 PMC3698523

[23] Kennedy E Rossi GR Vahanian NN Link CJ Phase 1/2 trial of the indoleamine 2,3-dioxygenase pathway (IDO) inhibitor indoximod plus ipilimumab for the treatment of unresectable stage 3 or 4 melanoma. ASCO Meet Abstr (2014) 32:TS9117.

[24] Gibney GT Hamid O Gangadhar TC Lutzky J Olszanski AJ Gajewski T Preliminary results from a phase 1/2 study of INCB024360 combined with ipilimumab (ipi) in patients (pts) with melanoma. ASCO Meet Abstr (2014) 32:3010.

[25] Jha GG Miller JS A randomized, double-blind phase 2 study of sipuleucel-T followed by indoximod or placebo in the treatment of patients with asymptomatic or minimally symptomatic metastatic castration-resistant prostate cancer. ASCO Meet Abstr (2014) 32:TS5111.

[26] Soliman HH Minton SE Ismail-Khan R Han HS Janssen W Vahanian NN A phase 2 study of Ad.p53 DC vaccine in combination with indoximod in metastatic solid tumors. ASCO Meet Abstr (2014) 32:TS3125.

[27] Kalinski P. Regulation of immune responses by prostaglandin E2. J Immunol (2012) 188:21–8. 10.4049/jimmunol.1101029 22187483 PMC3249979

[28] Lanzinger M Jurgens B Hainz U Dillinger B Raberger J Fuchs D Ambivalent effects of dendritic cells displaying prostaglandin E2-induced indoleamine 2,3-dioxygenase. Eur J Immunol (2012) 42:1117–28. 10.1002/eji.201141765 22539287

[29] Prendergast GC. Immune escape as a fundamental trait of cancer: focus on IDO. Oncogene (2008) 27:3889–900. 10.1038/onc.2008.35 18317452

[30] Shoushtari AN D’Angelo SP Keohan ML Dickson MA Gounder MM Abdullah AK Combined KIT and CTLA-4 blockade in patients with refractory GIST and other advanced sarcomas. ASCO Meet Abstr (2014) 32:10521. 10.1158/1078-0432.CCR-16-2349 PMC5486863 28007774

[31] Johnston PA Grandis JR. STAT3 signaling: anticancer strategies and challenges. Mol Interv (2011) 11:18–26. 10.1124/mi.11.1.4 21441118 PMC3063716

[32] Orabona C Pallotta MT Volpi C Fallarino F Vacca C Bianchi R SOCS3 drives proteasomal degradation of indoleamine 2,3-dioxygenase (IDO) and antagonizes IDO-dependent tolerogenesis. Proc Natl Acad Sci U S A (2008) 105:20828–33. 10.1073/pnas.0810278105 19088199 PMC2634889

[33] Iversen TZ Engell-Noerregaard L Ellebaek E Andersen R Larsen SK Bjoern J Phase I study of peptide vaccine targeting indeolamine 2,3 dioxygenase in metastatic lung cancer patients. ASCO Meet Abstr (2013) 31:8084. 10.1158/1078-0432.CCR-13-1560 24218513

[34] Opitz CA Litzenburger UM Sahm F Ott M Tritschler I Trump S An endogenous tumour-promoting ligand of the human aryl hydrocarbon receptor. Nature (2011) 478:197–203. 10.1038/nature10491 21976023

[35] Salter M Hazelwood R Pogson CI Iyer R Madge DJ. The effects of a novel and selective inhibitor of tryptophan 2,3-dioxygenase on tryptophan and serotonin metabolism in the rat. Biochem Pharmacol (1995) 49:1435–42. 10.1016/0006-2952(95)00006-L 7539265

[36] Dolusic E Larrieu P Moineaux L Stroobant V Pilotte L Colau D Tryptophan 2,3-dioxygenase (TDO) inhibitors. 3-(2-(pyridyl)ethenyl)indoles as potential anticancer immunomodulators. J Med Chem (2011) 54:5320–34. 10.1021/jm2006782 21726069

[37] Kanai M Funakoshi H Takahashi H Hayakawa T Mizuno S Matsumoto K Tryptophan 2,3-dioxygenase is a key modulator of physiological neurogenesis and anxiety-related behavior in mice. Mol Brain (2009) 2:8. 10.1186/1756-6606-2-8 19323847 PMC2673217

[38] Pantouris G Mowat CG. Antitumour agents as inhibitors of tryptophan 2,3-dioxygenase. Biochem Biophys Res Commun (2014) 443:28–31. 10.1016/j.bbrc.2013.11.037 24269239

[39] Ott M Litzenburger UM Rauschenbach KJ Bunse L Ochs K Sahm F Suppression of TDO-mediated tryptophan catabolism in glioblastoma cells by a steroid-responsive FKBP52-dependent pathway. Glia (2014) 63:78–90. 10.1002/glia.22734 25132599

[40] Wincent E Bengtsson J Mohammadi Bardbori A Alsberg T Luecke S Rannug U Inhibition of cytochrome P4501-dependent clearance of the endogenous agonist FICZ as a mechanism for activation of the aryl hydrocarbon receptor. Proc Natl Acad Sci U S A (2012) 109:4479–84. 10.1073/pnas.1118467109 22392998 PMC3311358

[41] Zelante T Iannitti RG Cunha C De Luca A Giovannini G Pieraccini G Tryptophan catabolites from microbiota engage aryl hydrocarbon receptor and balance mucosal reactivity via interleukin-22. Immunity (2013) 39:372–85. 10.1016/j.immuni.2013.08.003 23973224

[42] Moura-Alves P Fae K Houthuys E Dorhoi A Kreuchwig A Furkert J AhR sensing of bacterial pigments regulates antibacterial defence. Nature (2014) 512:387–92. 10.1038/nature13684 25119038

[43] Li Y Innocentin S Withers DR Roberts NA Gallagher AR Grigorieva EF Exogenous stimuli maintain intraepithelial lymphocytes via aryl hydrocarbon receptor activation. Cell (2011) 147:629–40. 10.1016/j.cell.2011.09.025 21999944

[44] Quintana FJ Basso AS Iglesias AH Korn T Farez MF Bettelli E Control of T(reg) and T(H)17 cell differentiation by the aryl hydrocarbon receptor. Nature (2008) 453:65–71. 10.1038/nature06880 18362915

[45] Esser C Rannug A Stockinger B. The aryl hydrocarbon receptor in immunity. Trends Immunol (2009) 30:447–54. 10.1016/j.it.2009.06.005 19699679

[46] Moennikes O Loeppen S Buchmann A Andersson P Ittrich C Poellinger L A constitutively active dioxin/aryl hydrocarbon receptor promotes hepatocarcinogenesis in mice. Cancer Res (2004) 64:4707–10. 10.1158/0008-5472.CAN-03-0875 15256435

[47] Bessede A Gargaro M Pallotta MT Matino D Servillo G Brunacci C Aryl hydrocarbon receptor control of a disease tolerance defence pathway. Nature (2014) 511:184–90. 10.1038/nature13323 24930766 PMC4098076

[48] Mezrich JD Fechner JH Zhang X Johnson BP Burlingham WJ Bradfield CA. An interaction between kynurenine and the aryl hydrocarbon receptor can generate regulatory T cells. J Immunol (2010) 185:3190–8. 10.4049/jimmunol.0903670 20720200 PMC2952546

[49] Munn DH Sharma MD Baban B Harding HP Zhang Y Ron D GCN2 kinase in T cells mediates proliferative arrest and anergy induction in response to indoleamine 2,3-dioxygenase. Immunity (2005) 22:633–42. 10.1016/j.immuni.2005.03.013 15894280

[50] Sharma MD Baban B Chandler P Hou DY Singh N Yagita H Plasmacytoid dendritic cells from mouse tumor-draining lymph nodes directly activate mature Tregs via indoleamine 2,3-dioxygenase. J Clin Invest (2007) 117:2570–82. 10.1172/JCI31911 17710230 PMC1940240

[51] Munn DH Shafizadeh E Attwood JT Bondarev I Pashine A Mellor AL. Inhibition of T cell proliferation by macrophage tryptophan catabolism. J Exp Med (1999) 189:1363–72. 10.1084/jem.189.9.1363 10224276 PMC2193062

[52] Kudo Y Boyd CA. The role of l-tryptophan transport in l-tryptophan degradation by indoleamine 2,3-dioxygenase in human placental explants. J Physiol (2001) 531:417–23. 10.1111/j.1469-7793.2001.0417i.x 11230514 PMC2278460

[53] Haynes BF Hemler ME Mann DL Eisenbarth GS Shelhamer J Mostowski HS Characterization of a monoclonal antibody (4F2) that binds to human monocytes and to a subset of activated lymphocytes. J Immunol (1981) 126:1409–14. 7204970

[54] Fenczik CA Sethi T Ramos JW Hughes PE Ginsberg MH. Complementation of dominant suppression implicates CD98 in integrin activation. Nature (1997) 390:81–5. 10.1038/36349 9363894

[55] Seymour RL Ganapathy V Mellor AL Munn DH. A high-affinity, tryptophan-selective amino acid transport system in human macrophages. J Leukoc Biol (2006) 80:1320–7. 10.1189/jlb.1205727 16997853

[56] Kaper T Looger LL Takanaga H Platten M Steinman L Frommer WB. Nanosensor detection of an immunoregulatory tryptophan influx/kynurenine efflux cycle. PLoS Biol (2007) 5:e257. 10.1371/journal.pbio.0050257 17896864 PMC1988858

